# Master Regulatory Transcription Factors in β-Aminobutyric Acid-Induced Resistance (BABA-IR): A Perspective on Phytohormone Biosynthesis and Signaling in *Arabidopsis thaliana* and *Hordeum vulgare*

**DOI:** 10.3390/ijms25179179

**Published:** 2024-08-23

**Authors:** Eszter Virág, Ágnes Nagy, Beáta B. Tóth, Barbara Kutasy, József Péter Pallos, Zsuzsa Máthéné Szigeti, Csaba Máthé, Gábor Kardos, Géza Hegedűs

**Affiliations:** 1 Research Institute for Medicinal Plants and Herbs Ltd., 2011 Budakalász, Hungary; aginagy.nagy@gmail.com (Á.N.); kutasy.barbara.julia@uni-mate.hu (B.K.); pallos.jp@gynki.hu (J.P.P.); mzsuzsa768@gmail.com (Z.M.S.); 2 Institute of One Health, Faculty of Health Science, University of Debrecen, Egyetem Tér 1, H-4032 Debrecen, Hungary; toth.beata@etk.unideb.hu (B.B.T.); kg@med.unideb.hu (G.K.); 3 Department of Plant Physiology and Plant Ecology, Institute of Agronomy, Hungarian University of Agriculture and Life Sciences, Georgikon Campus, Festetics Str 7, 8360 Keszthely, Hungary; 4 Department of Botany, Faculty of Science and Technology, University of Debrecen, Egyetem Tér 1, H-4032 Debrecen, Hungary; mathe.csaba@science.unideb.hu; 5 Department of Information Technology and Its Applications, Faculty of Information Technology, University of Pannonia, Gasparich Márk Str. 18/A, 8900 Zalaegerszeg, Hungary

**Keywords:** induced resistance, priming, β-aminobutyric acid (BABA), transcription factor, phytohormone, *cis*-acting factors, DOF, AHL, ERF

## Abstract

The endogenous stress metabolite β-aminobutyric acid (BABA) primes plants for enhanced resistance against abiotic and biotic stress by activating a complex phytohormone signaling network that includes abscisic acid (ABA), jasmonic acid (JA), salicylic acid (SA), and ethylene (ET). In this study, through stringent filtering, we identify 14 master regulatory transcription factors (TFs) from the DOF, AHL, and ERF families that potentially regulate the biosynthesis and signaling of these phytohormones. Transcriptional analysis of BABA-treated *Arabidopsis thaliana* and *Hordeum vulgare* suggests that DOF family TFs play a crucial role in stress response regulation in both species. BABA treatment in *A. thaliana* upregulates the TFs MNB1A and PBF and enhances the expression of the genes ICS1, EDS5, and WIN3 in the SA biosynthesis pathway, potentially boosting NPR1 and PR1 in the SA signaling pathway. Conversely, in *H. vulgare*, the BABA-induced upregulation of TF DOF5.8 may negatively regulate SA biosynthesis by downregulating ICS1, EDS5, and PR1. Additionally, in *A. thaliana*, BABA triggers the expression of TF PBF, which may result in the decreased expression of MYC2, a key gene in JA signaling. In contrast, *H. vulgare* exhibits increased expression of ERF2 TF, which could positively regulate the JA biosynthesis genes LOX and Tify9, along with the COI1 and JAZ genes involved in the JA signaling pathway. These findings offer new perspectives on the transcriptional regulation of phytohormones during plant priming.

## 1. Introduction

Plants often face recurrent biotic and abiotic stresses, which can lead to substantial yield losses, particularly under severe conditions. In addition to their inherent defense mechanisms, plants can activate structural and chemical defenses in response to pathogen attacks [1]. These inducible defenses are governed by the innate immune system of plants [2], offering protection against various potentially detrimental microorganisms [3]. The regulation of plant immunity is mediated by small-molecule hormones such as salicylic acid (SA), jasmonic acid (JA), abscisic acid (ABA), and ethylene (ET) [4,5,6,7]. 

The priming molecule β-aminobutyric acid (BABA) is naturally occurring in plants and is induced by stress, indicating its role as an endogenous stress metabolite [8,9]. As an isomer of γ-aminobutyric acid (GABA), a neurotransmitter in animals, BABA may also function as a signaling molecule in plants [10]. First identified in 1963 as a substance with potential plant-protective properties [11], research into its effects on induced resistance (IR) and underlying mechanisms began in the 1990s [12,13,14,15,16,17]. These studies have established BABA as a broad-spectrum agent that is highly effective in stimulating multiple defense pathways against both abiotic and biotic stresses. It has been shown to enhance plant resistance against up to 80 different pathogens and pests, including bacteria, fungi, insects, nematodes, and viruses, as well as environmental stresses such as drought, heat, cold, and high salinity [16,18,19,20,21,22,23,24,25,26].

Numerous studies have documented the enhancement of defense pathways triggered by BABA, including the direct stimulation of signaling pathways involving SA, JA, ET, ABA, and pathogenesis-related (PR) genes. The activation of these pathways is highly dependent on the specific type of stress and varies considerably depending on both the pathogen and the plant species [12,20,27,28]. BABA triggers several transcription factor (TF) families, including WRKY, which activates ICS1 and PBS3 promoters in the SA signaling pathway [29]. MYC and AP2/ERF regulate distinct branches of the JA signaling pathway, with MYC being co-regulated by ABA and AP2/ERF by ET [30]. However, there is limited information on how these different stress signals interact, how they are integrated into a complex response, and what regulatory elements control these processes. 

We hypothesized that distinct hormonal responses in plants might arise from BABA treatment in monocot and dicot cultures, even before exposure to stresses, with these responses potentially influenced by taxonomic characteristics. Furthermore, it was assumed that such master TFs could orchestrate hormonal regulation under inductive conditions like BABA-IR. 

The primary objective of our study is to identify potential transcriptional regulators (TFs) associated with genes involved in various stress hormone signaling pathways, including JA, SA, ABA, and ET, in plant systems. This is achieved by investigating the presence of TF response elements in the promoters of these genes and analyzing transcriptomic data from the BABA-treated monocot *Hordeum vulgare* and dicot *Arabidopsis thaliana*. By systematically screening the results, we narrow down the range of TFs that may serve as regulators within these stress hormone signaling cascades, distinguishing those that likely participate in single versus multiple transduction processes, thus influencing the plant’s overall stress response. Additionally, through a comparative analysis of differential gene expression profiles between BABA-treated barley and *Arabidopsis*, we identify candidate general and species-specific TFs that have response elements within the genes of these stress signaling pathways. Targeting these putative master regulators could enable more precise and controlled priming of plants against various stressors, thereby enhancing their defense mechanisms. 

## 2. Results

### 2.1. Pathway Analysis of DEGs Revealed Enhanced MAPK Signaling, Plant–Pathogen Interactions, Plant Hormone Signal Transduction, and Decreased Expression of Photosynthetic Genes in Both Species Following BABA Treatment

The analysis of differentially expressed genes (DEGs) from pairwise gene expression comparisons between BABA-treated and control *A. thaliana* and *H. vulgare* plants identified 927 and 619 upregulated genes, and 1419 and 1051 downregulated genes, respectively (data available at: https://data.mendeley.com/datasets/mkjrs4cjxk/1, accessed on 7 August 2024). Gene set enrichment analysis (GSEA) was conducted on the entire DEGs dataset, alongside pathway analysis. 

**Figure 1:**
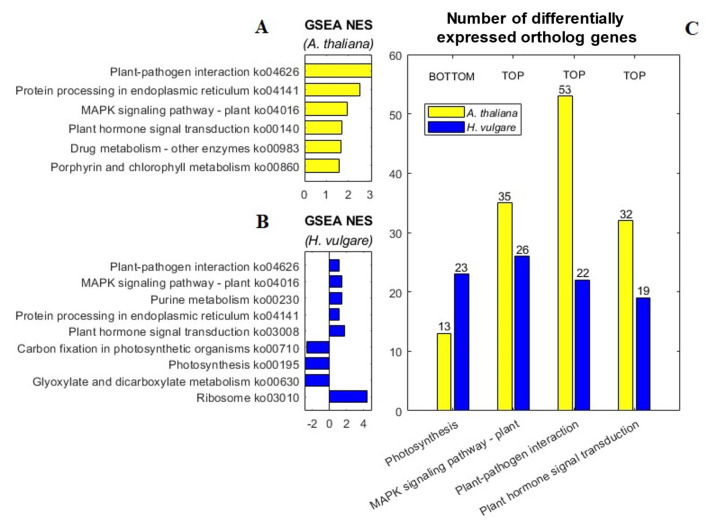
KEGG pathway analysis of differentially expressed genes (DEGs) from BABA-treated *A. thaliana* and *H. vulgare* identified the pathways with the most significant changes based on NES values, which are illustrated for *A. thaliana* (**A**) and *H. vulgare* (**B**). The pathways that showed consistent changes in both species (**C**) were further analyzed to identify the ortholog groups involved in these pathways.

**Figure 2:**
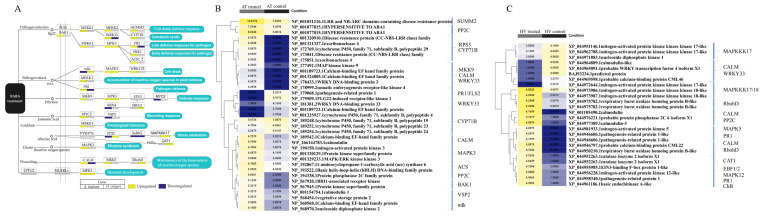
A pathway diagram (**A**) of ortholog groups mapped to the MAPK signaling KEGG pathway. Yellow and dark blue colors represent upregulated and downregulated groups, respectively, with light blue boxes highlighting the final output processes associated with the mapped ortholog genes. These processes include cell death, camalexin biosynthesis, defense-related gene induction, wound response, stress adaptation, ethylene biosynthesis, redox homeostasis, and stomatal development. Differentially expressed orthologs in *A. thaliana* (**B**) and *H. vulgare* (**C**) are visualized using heat maps. Hierarchical clustering, based on the Euclidean distance between genes, was used to generate the dendrograms displayed on the left. Expression data were calculated using the logarithm of CPM.

**Figure 3:**
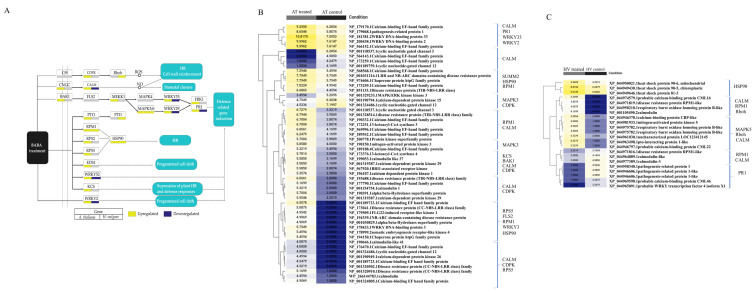
A pathway diagram (**A**) of ortholog groups mapped to the plant–pathogen interaction KEGG pathway. Yellow and dark blue colors represent upregulated and downregulated ortholog groups, respectively, with light blue boxes highlighting the final output processes associated with the mapped genes. These processes include cell wall reinforcement, stomatal closure, defense-related gene induction, hypersensitive reaction (HR), programmed cell death, and the suppression of plant HR and defense responses. Differentially expressed ortholog genes in *A. thaliana* (**B**) and *H. vulgare* (**C**) are visualized using heatmaps. Hierarchical clustering, based on the Euclidean distance between genes, was used to generate the dendrograms displayed on the left. Expression data were determined using the logarithm of CPM.

**Figure 4:**
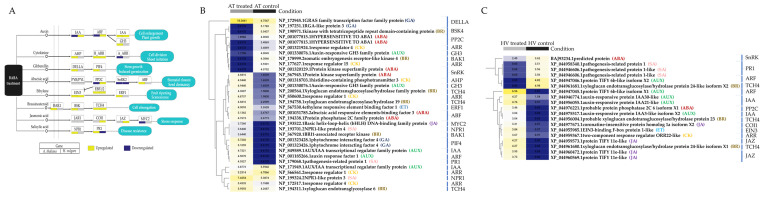
A pathway diagram (**A**) illustrating ortholog groups mapped to the plant hormone signal transduction KEGG pathway. Yellow and dark blue colors represent upregulated and downregulated ortholog groups, respectively. Final output processes associated with mapped genes are marked with light blue boxes. These processes include cell enlargement, plant growth, cell division, shoot initiation, stem growth, induced germination, stomatal closure, seed dormancy, fruit ripening, senescence, cell elongation, stress response, and disease resistance. Differentially expressed genes (DEGs) grouped by hormonal impact in *A. thaliana* (**B**) and *H. vulgare* (**C**) are visualized on heat maps. A hierarchical clustering method, utilizing the Euclidean distance between genes, was applied to produce the dendrograms on the left. Expression data were determined by the logarithm of CPM.

According to the top GSEA Normalized Enrichment Score (NES) values of pathways, the BABA treatment revealed distinct Kyoto Encyclopedia of Genes and Genomes (KEGG) pathways with significant changes: a decrease in photosynthesis-related pathways and an increase in pathways associated with Mitogen-activated protein kinase (MAPK) signaling, plant–pathogen interactions, and plant hormone signal transduction in both species. The number of ortholog groups (each comprising a full set of functionally related genes) mapped to these pathways was greater in *A. thaliana* (Figure 1). The analysis of the expression patterns of these ortholog groups highlighted a marked difference between *A. thaliana* and *H. vulgare*, allowing us to identify altered physiological responses linked to these pathways (Figure 2, Figure 3 and Figure 4). Subsequently, we examined the complete set of genes from KEGG hits that constitute similar functional groups (referred to as “ortholog genes”). We conducted a pairwise expression analysis of these genes to obtain a clearer understanding of species-specific responses. The results are presented in heatmaps (Figure 2, Figure 3 and Figure 4). 

Phenotypic alterations were found to indicate decreased photosynthetic activity in *A. thaliana* after BABA treatment from 48 h (see Section 4.1 Plant materials paragraph).

#### 2.1.1. BABA Treatment Improved Oxidative Homeostasis in *H. vulgare* and Enhanced Pathogen Response in *A. thaliana* through Activation of MAPK Signaling Pathway

We identified 35 ortholog genes in *A. thaliana* and 26 ortholog genes in *H. vulgare* associated with 32 ortholog groups mapped to the MAPK signaling pathway (Figure 2). Analyzing their expression profiles, we evaluated the impact of BABA treatment on these signaling processes in both plant species (Figure 2A). Notably, the expression of *A. thaliana* orthologs (based on The Arabidopsis Information Resource—TAIR) differed significantly between the two species. 

The brassinosteroid-insensitive 1-associated receptor kinase 1 (BAK1, AT4G33430), a serine/threonine protein kinase, was upregulated in *A. thaliana* and triggered several gene cascades (Figure 2B). These cascades include the following: (i) mitogen-activated protein kinase kinase 1 (MEKK1) (AT4G08500), MEKK2 (AT4G08480), and the suppressor of MAP kinase kinase 1 (MKK1) and 2 (disease resistance protein 2—SUMM2, AT1G12280), which may initiate cell death and defense responses to bacteria; (ii) WRKY33 (AT2G38470) and CYP71B15 (AT3G26830), which are involved in camalexin biosynthesis; and (iii) MPK3 (AT3G45640), pathogenesis-related protein 1 (PR1, AT2G14610), FLG22-induced receptor-like kinase (FRK1, AT2G19190), and 1-aminocyclopropane-1-carboxylate synthase (ACS1, AT3G61510), which contribute to early and late defense responses to pathogens and ethylene synthesis.

In contrast, JA-mediated anti-insect activity processes were suggested to be downregulated in *A. thaliana* through Vegetative Storage Protein 2 (VSP2, AT5G24770), regulated by the transcription factor MYC2 (AT1G32640), with no corresponding changes observed in *H. vulgare*. Notably, in *H. vulgare*, BABA treatment led to the upregulation of (i) signaling pathways involved in maintaining redox balance through respiratory burst oxidase protein D (RbohD, AT5G47910) and catalase (CAT1, AT1G20630) and (ii) PP2C family proteins associated with stress adaptation (Figure 2C).

#### 2.1.2. After BABA Treatment, the Gene Signaling Cascade Regulated by the WRKY Family Was Upregulated in *A. thaliana* but Downregulated in *H. vulgare* as Part of the Plant–Pathogen Interaction Pathway

We identified 53 ortholog genes in *A. thaliana* and 22 in *H. vulgare* linked to 21 ortholog groups mapped to the plant–pathogen interaction pathway (Figure 3). In both species, the hypersensitive response was mediated by resistance to the *Pseudomonas syringae* gene (RPM1, AT3G07040) cascade, which involved heat shock protein 90 (HSP90, AT5G52640). Within this cascade, the genes resistant to *P. syringae* 2 and 5 (RPS2, AT4G26090; RPS5, AT1G12220) showed elevated expression only in *A. thaliana*. In contrast, cytosolic ABA receptor kinase (PTI1, AT2G47060) and Rboh family proteins were upregulated in *H. vulgare*, suggesting that the hypersensitive response may be more pronounced through this alternate pathway in response to BABA treatment in barley. Additionally, members of the WRKY transcription factor family (WRKY2/52/29/33, AT5G56270, AT1G69310, AT4G23550, AT2G38470) were upregulated in *A. thaliana* but downregulated in *H. vulgare*. Furthermore, the enhanced disease susceptibility 1 (EDS1, AT3G48090) gene, which is involved in programmed cell death, was upregulated in *A. thaliana.*

#### 2.1.3. BABA Treatment Activated Distinct Phytohormone Signal Transduction Cascades in *A. thaliana* and *H. vulgare*, Leading to the Induction of Defense-Related Genes

We identified 32 ortholog genes in *A. thaliana* and 19 in *H. vulgare* linked to 20 ortholog groups mapped to the plant hormone signal transduction pathway (Figure 4). In this pathway, the auxin (AUX), ET, JA, and ABA signaling pathways were upregulated in *H. vulgare*, while the brassinosteroid (BR), SA, gibberellin (GA), and cytokinin (CK) signaling pathways were upregulated in *A. thaliana*. Key genes in the AUX signaling pathway, such as the AUX-induced protein family (IAA, AT1G19220) and the AUX responsive factor (ARF) family, were upregulated in *H. vulgare* but downregulated in *A. thaliana*. The Gretchen Hagen acyl acid amido synthetase (GH3, AT5G13370) was overexpressed in both species, regulating free hormone concentration and downstream responses.

In both species, the cell-to-cell mobile RNA Ein3-binding F-box protein 1/2 (EBF1/2, AT2G25490, AT5G25350) was upregulated, associated with the ET pathway. The JA signaling pathway showed upregulation of the coronatine insensitive 1 (COI1, AT2G39940) gene, which interacts with JAZ family genes, indicating JA stimuli in *H. vulgare*. The ABA signaling pathway was downregulated in *A. thaliana* due to the downregulation of type 2C protein phosphatases (PP2C family), which are standard negative regulators of ABA signaling, along with the kinases of SnRK2 (AT3G50500). In contrast, in *H. vulgare*, PP2C ortholog genes were upregulated, suggesting inhibition of ABA signaling. 

In CK signaling, the essential genes include the *Arabidopsis* response regulator (ARR) complex components. The type-B ARR family genes were upregulated in both species, while the type-A ARR family genes were downregulated in *A. thaliana* and unaffected in *H. vulgare*. Positive regulators of CK signaling, such as the *A. thaliana* histidine phosphotransfer proteins (AHPs), were upregulated only in *A. thaliana* and showed no change in *H. vulgare*.

The GA pathway displayed upregulation of genes mapped to the GRAS family transcription factor DELLA and the phytochrome interacting factor (PIF4, AT2G43010) exclusively in *A. thaliana*. In BR signaling, the kinases BSK1 (AT4G35230) and BAK1 (AT4G33430) were upregulated in *A. thaliana*, leading to the upregulation of the cell wall-modifying enzyme xyloglucan:xyloglucosyl transferase TCH4 (AT5G57560), indicating a rapid response to environmental stimuli. In *H. vulgare*, only TCH4 was overexpressed, suggesting its upregulation is independent of brassinosteroid signaling.

For the SA signaling pathway, NPR1 and PR1 were upregulated in *A. thaliana*, whereas PR1 was downregulated in *H. vulgare*.

### 2.2. DEGs Revealed Species-Specificity of ABA, SA, JA, and ET Metabolism Genes, Highlighting Distinct Biosynthetic Pathways Activated Following BABA Treatment

**Figure 5:**
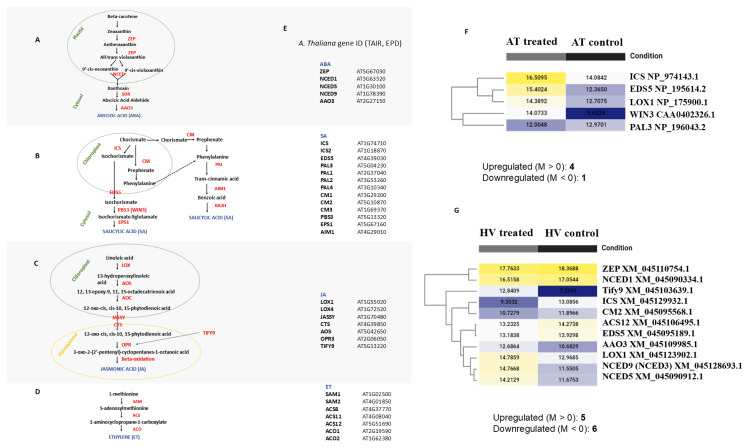
(**A**–**D**) Biosynthetic steps of abscisic acid (ABA) (**A**), salicylic acid (SA) (**B**), jasmonic acid (JA) (**C**), and ethylene (ET) (**D**) in *A. thaliana*, with the corresponding list of genes catalyzing these biosynthesis processes presented alongside TAIR and EPD IDs (**E**). Pairwise expression analysis was conducted using orthologs of TAIR genes identified in *A. thaliana* and *H. vulgare* genomes. The results are displayed on heat maps, indicating the genomic IDs (**F**,**G**). A total of 32 genes were analyzed, resulting in 5 differentially expressed genes (DEGs) in *A. thaliana* (**F**) and 11 DEGs in *H. vulgare* (**G**). The logarithm of CPM was used to determine expression differences.

We analyzed 32 *A. thaliana* genes involved in the biosynthetic pathways of ABA, SA, JA, and ET (Figure 5A–D). These genes, identified by their TAIR IDs (Figure 5E), were compared to their orthologs in *H. vulgare* to conduct a pairwise expression analysis. The investigated TAIR genes are well-documented in BABA-priming research.

The pairwise expression analysis revealed distinct hormonal regulatory mechanisms between the two species (Figure 5F,G). *H. vulgare* exhibited a slightly higher number of DEGs compared to *A. thaliana*, with five upregulated and six downregulated genes, and four upregulated and one downregulated gene, respectively.

In *A. thaliana*, significant upregulation was observed in genes such as isochorismate synthase (ICS), hopw1-1-interacting3 (WIN3), lipoxygenase 1 (LOX1), and enhanced disease susceptibility5 (EDS5), while phenylalanine ammonia-lyase (PAL) was downregulated. Conversely, in *H. vulgare*, the regulation differed, with upregulated genes including lipoxygenase (LOX1), jasmonate-associated1 (Jaz10 or Tify9), nine-cis-epoxycarotenoid dioxygenase (NCED5/9), and aldehyde oxidase (AAO3). Strong downregulation was noted for isochorismate synthase (ICS), chorismate mutase (CM2), 1-aminocyclopropane-1-carboxylate synthase12 (ACS12), EDS5, carotenoid cleavage dioxygenase (NCED1), and zeaxanthin epoxidase (ZEP). The downregulation of PAL3 in *A. thaliana* suggests that the prephenate branch of SA biosynthesis may become less significant following BABA treatment. Conversely, in *H. vulgare*, the downregulation of ICS and CM2 indicates a suppression of SA biosynthesis early in the pathway, which, along with the downregulation of EDS5, may redirect SA synthesis through the phenylalanine branch. Additionally, the downregulation of ZEP in *H. vulgare* points to a disrupted ABA biosynthesis pathway, while the JA biosynthetic process remains unaffected and continues to be upregulated.

In silico results were validated by selecting six genes of interest in *A. thaliana*—AAO3, ZEP, OPR1, AOC1, PAL3, and ICS1—and measuring their relative expression using RT-qPCR. The relative expression values demonstrated a strong correlation (R = 0.996249) with the RPM (Reads per million mapped reads) index of these genes. The data supporting this correlation are presented in Figure S1.

### 2.3. Identification of Jaspar Core TF Binding Motifs in the Promoters of Genes Involved in Phytohormone Biosynthesis and Signaling, and Their Expression Analysis following BABA Treatment

#### 2.3.1. Screening of Master Regulatory TFs Involved in Phytohormone Signaling Pathways

Differential expression analysis of BABA-treated *Hordeum* sp. and *Arabidopsis* sp. suggests that several TFs, such as WRKYs, MYCs, DELLAs, and JAZs, may function as downstream regulators in BABA-induced stress signaling across the SA, JA, ABA, and ET pathways of the phytohormone network. We hypothesized that the TF-regulated biosynthesis of phytohormones may exhibit species-specific characteristics. To explore this hypothesis further, we focused on identifying master regulatory TFs that occupy key positions within a regulatory hierarchy, orchestrating the entire hormonal network in stress responses. These master TFs were defined based on their ability to bind to the promoters of genes involved in stress hormone biosynthesis and signaling pathways, thereby modulating the expression of these genes.

**Scheme 1:**
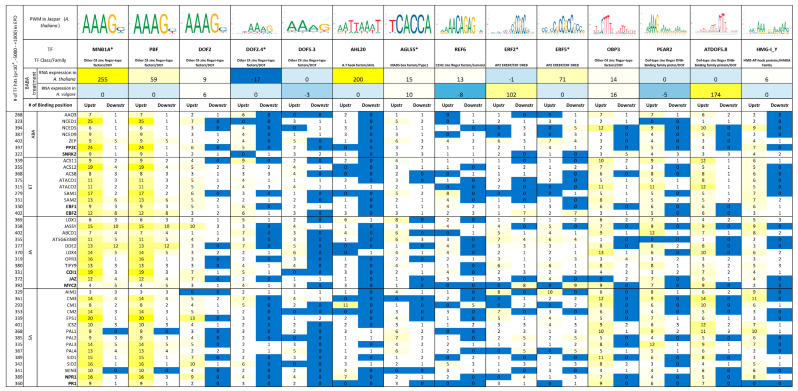
Master regulatory transcription factors of the investigated biosynthetic and signaling genes. The total number of possible binding TFs of each investigated gene screened in the EPD are presented. The sequence logo representation of the DNA binding specificity of the transcription factors is presented. The hits of upstream and downstream positions are presented in the investigated gene promoters, with a range between −5000 and +1000, with the stringency of *p* < 1 × 10^−4^ outcomes. No hits (0) are colored with dark blue and increases in hits are indicated by the intensity of the yellow. Changes in RNA expression levels in masters (CPM, in %) are marked as follows: downregulated: dark blue; no changes: light blue; moderately upregulated: not colored; upregulated: yellow. Signaling genes are marked with bold, and biosynthetic genes with smooth. Shared-signaling biosynthetic master regulatory TFs are marked with stars.

Promoter information for 32 biosynthetic genes of *A. thaliana* involved in the DEG analysis was retrieved from the Eukaryotic Promoter Database (EPD) (Figure 5E). We focused on TFs with a high probability of binding to motifs located within the upstream and downstream regions of the DNA sequence (−5000 to +1000 bp), applying a stringency threshold of *p* < 1 × 10^−4^ (data available at: https://data.mendeley.com/datasets/mkjrs4cjxk/1, accessed on 18 August 2024). This analysis identified 14 master regulatory TFs associated with the biosynthetic genes of the SA, JA, ABA, and ET pathways. Notably, 7 of these 14 TFs were also determined to be master regulators of the ABA, SA, JA, and ET signaling pathways (Scheme 1). 

A total of four SA, 9 JA, 5 ABA, and ten ET biosynthetic master regulatory TFs were identified, belonging to the following six TF families: (i) DNA-binding One Zinc Finger, DOF (MNB1A, PBF, DOF2, DOF2.4, DOF5.3, DOF3.6, OBP3, DOF5.1, DOF5.8); (ii) AT-hook motif nuclear localized, AHL (AHL20); (iii) MADS-box Type1 (AGL55); (iv) Jumonji (REF6); (v) Ethylene Response Factors and Dehydration-Responsive Element-Binding, ERF/DREB (ERF2, ERF5); and (vi) High Mobility Group A, HMGA (HMGI_Y). The known functions of these 14 master regulatory TFs are summarized in Table 1. 

**Figure 6:**
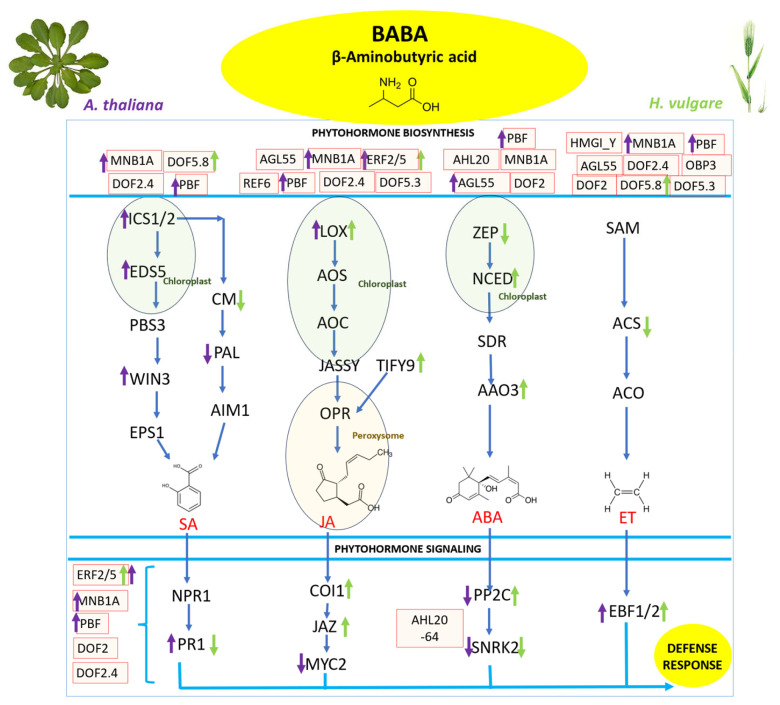
The schematic illustration of the impact of BABA treatment on the biosynthesis and signaling pathways of SA, JA, ABA, and ET, as well as their regulation by master TFs. Purple and green arrows denote the upregulation and downregulation of genes in *A. thaliana* and *H. vulgare*, respectively. In the SA signaling pathway, distinct regulatory roles of ERF TFs were observed in *A. thaliana* and *H. vulgare*, particularly affecting the PR1 gene. BABA treatment negatively modulates ABA signaling via the PP2C/SNRK2 pathway, with AHL20 binding at the -64 promoter position in *A. thaliana*. Additionally, BABA treatment enhances the expression of EBF1/2, which acts as a negative regulator in the ET signaling pathway in both plant species, thereby repressing ET-related responses.

Additionally, nine genes involved in phytohormone signaling (Figure 6) that exhibited altered expression were included in the search for TF promoter binding sites using the *A. thaliana* query in the EPD (Scheme 1).

Scheme 1 summarizes information on the TFs associated with these genes, including the total number of TF binding site hits, the position weight matrix (PWM) of master regulatory TFs, and the number of upstream and downstream binding regions in the master promoters.

#### 2.3.2. An Analysis of Promoter Binding Sites Based on the Position Frequency of Master Regulatory TFs

The total number of potential TF binding sites, based on the PWM, and the number of binding sites for master TFs in both upstream and downstream regions are summarized in Scheme 1. The promoters of the ZEP and EBF2 genes exhibited the highest number of TF hits, with a total of 402, whereas the AAO3 gene promoter had the fewest, with 268 hits. Notably, the highest binding site frequency in upstream and downstream regions was observed for MNB1A and PBF, both of which were upregulated in *Arabidopsis*. Interestingly, the DNA binding domains of MNB1A and PBF were found to be identical.

Additionally, DOF5.8 showed a higher frequency of binding across all investigated gene promoters that were upregulated in *Hordeum* following BABA treatment. While OBP3 and phloem early dof 2 (PEAR2) also demonstrated high binding frequencies among master TFs, no significant expression changes were observed in response to BABA treatment. In particular, there was a notable absence of downstream binding sites in the promoters of PAL1 and WIN3, which are associated with the SA biosynthetic pathway. Furthermore, the lack of downstream binding sequences for AHL20 was consistently observed across all investigated genes.

#### 2.3.3. Upregulation of DOF, AHL, and ERF Families as Master Regulatory TFs in *A. thaliana* and *H. vulgare* Following BABA Treatment

Gene expression analysis of the 14 master TFs revealed the significant upregulation of members from the DOF, AHL, and ERF TF families in response to BABA treatment. Among these, the DOF family emerged as the most prominent master regulator of the investigated phytohormone pathways. Notable increases in expression were observed in DOF family members, including MNB1A, which showed a 225% increase in CPM in *A. thaliana*, and DOF5.8, which exhibited a 174% increase in CPM in *H. vulgare*. Additionally, AHL20 demonstrated a 200% increase in gene expression in *A. thaliana*. The ERF family members ERF2 and ERF5 also showed upregulation, with increases of 102% and 71% in CPM, respectively. Interestingly, the expression of MNB1A, PBF, AHL20, and ERF5 was notably elevated in *A. thaliana* following BABA treatment. These findings highlight the key roles of these TF families in regulating phytohormone signaling pathways and their altered expression profiles in response to stress. A summary of the phytohormone regulatory processes and their expression changes is illustrated in Figure 6.

The following relationships were observed between the expression of phytohormone biosynthetic and signaling genes and their promoter-binding master TFs after BABA treatment:

In *A. thaliana:* (i) The upregulation of the master TFs MNB1A and PBF correlated with the upregulation of genes involved in the isochorismate branch of the SA biosynthetic pathway, while there was a downregulation of genes in the PAL branch of the SA pathway. (ii) The NPR1 and PR1 genes, part of the SA signaling pathway, were both upregulated. (iii) The upregulation of ERF2, MNB1A, and PBF was associated with the upregulation of the LOX gene in the JA biosynthetic pathway, while the MYC2 gene involved in JA signaling was downregulated. (iv) ERF2, MNB1A, and PBF positively influenced the expression of the PP2C and SnRK2 genes involved in ABA signaling, as well as the EBF1/2 genes involved in ET signaling. (v) The upregulation of the master TF AHL20 was linked to the downregulation of the SNRK2 gene in the ABA signaling pathway. Notably, AHL20 was found to bind specifically to the promoter of SNRK2 among the signaling genes.

These findings highlight the intricate regulatory networks involving master TFs and their impact on phytohormone pathways following BABA treatment. 

In *H. vulgare*: (i) The co-expression of the master TFs ERF2 and DOF5.8 was observed, with these TFs regulating genes involved in JA, SA, and ET biosynthesis. However, no upregulated master TF associated with ABA biosynthesis was identified in *H. vulgare*. (ii) The upregulation of ERF2 was associated with the upregulation of the Tify9 and LOX genes involved in JA biosynthesis, as well as the COI1 and JAZ genes involved in JA signaling. (iii) The upregulation of DOF5.8 was linked with the downregulation of the ACS gene involved in ET biosynthesis. Additionally, DOF5.8 upregulation was associated with the downregulation of the ICS2, EDS5, and CM genes involved in SA biosynthesis.

These observations indicate the role of specific master TFs in modulating phytohormone pathways in *H. vulgare* following BABA treatment.

## 3. Discussion

The BABA treatment led to a significant enrichment in the KEGG pathways associated with plant–pathogen interactions, MAPK signaling, and plant hormone signal transduction. These findings are consistent with those reported by Zapletalová et al. [45] in their study on tomato plants.

These pathways varied between the investigated species, a phenomenon previously noted by Baccelli et al. (2016) [46]. Numerous studies have explored the impact of BABA on phytohormone genes across different plants, revealing several contradictions. For instance, research by Zapletalová et al. [45] suggested that SA was not implicated in the BABA signaling pathway in tomato, whereas Ren et al. reported increased expression of SA, JA, and ET-related genes following BABA treatment in tobacco [47]. These discrepancies may be attributed to the distinct taxonomic characteristics and associated genomic regulatory mechanisms of different plant species. These altered data may be due to the different taxonomic properties involving different genomic regulations. 

Therefore, this study addresses a previously underexplored aspect of phytohormone regulation in BABA priming, specifically focusing on the initial steps involving transcription factors and the genomic structures of the examined genes. We present novel findings that reveal previously unreported correlations in the regulation of ET, ABA, SA, and JA pathways. These insights contribute new understanding to the scientific literature on the role of transcription factors in BABA-induced phytohormone signaling.

### 3.1. The BABA Treatment May Stimulate the Negative Regulator EBF1/2 of ET Signal in Both Plants

ET production is an early plant response to pathogen attack [48]. However, our results indicate that BABA treatment did not activate the genes associated with the ET biosynthetic pathway in the plants studied. Despite this, we observed an upregulation of EBF1/2 transcripts, which are involved in the ubiquitin/proteasome-dependent degradation of ethylene insensitive3 (EIN3) protein. This finding aligns with the results of Zapletalová et al., who reported species-specific responses in ET signaling, noting that while ET signaling was not activated in *Arabidopsis*, it was in tomato [45]. EBF1/2 transcription is known to provide negative feedback on ET signaling by regulating EIN3, which can bind to EBF1/2 promoters and inhibit its own signaling [49,50,51].

In our study, we identified new master transcription factors, MNB1A and PBF, as potential negative regulators of ET signaling in *Arabidopsis*. Despite the upregulation of these negative regulators, no significant changes were observed in the ET biosynthetic and signaling pathways. This suggests that BABA treatment does not activate ET pathway genes but rather maintains a longer leaf lifespan through repression mechanisms. A similar phenomenon was described by Sós-Hegedűs et al. [52]. In *H. vulgare*, we observed the downregulation of the ACS gene alongside the upregulation of DOF5.8. Given that the overexpression of DOF5.8 correlated with the downregulation of the CM and ACS genes involved in SA and ET biosynthesis, we propose that DOF5.8 may function as a repressor in the transcription of phytohormone biosynthesis, a repression potentially triggered by BABA.

### 3.2. BABA Treatment May Negatively Regulate the ABA Signaling Genes PP2C and SNRK2, Potentially through the Action of Master Transcription Factors AHL20, PBF, and MNB1A

Our results indicate that BABA treatment leads to the downregulation of ABA signaling in *A. thaliana*, as evidenced by the reduced expression of PP2C and SNRK2 genes. AHL20, identified as a master transcription factor, appears to specifically regulate the ABA signaling pathway. Despite this, we did not observe changes in the ABA biosynthetic genes following BABA treatment. Notably, AHL20 was found to bind specifically to the promoter of the SNRK2 gene, suggesting a targeted negative regulation of this gene in *A. thaliana*. This finding is consistent with the work of Lu et al., who highlighted AHL20’s role in negatively regulating ABA-dependent defense mechanisms in *A. thaliana*, where the PP2C-SnRK2 complex plays a central role in innate immune responses [36,53]. While AHL20 has been linked to ABA control and signaling in monocots, our results show that AHL20 upregulation is specific to the dicot *A. thaliana* [54,55]. 

The transcription factor PBF, known for encoding an ABA-responsive element-binding protein, is typically expressed in response to stress and ABA. In litchi, PBF upregulates genes such as SNRK2 and PP2C [56]. However, our study indicates that PBF exerts a negative regulatory role on these genes in *A. thaliana*, suggesting a species-specific divergence in its function. 

MNB1A, identified as a DOF1 transcription factor [57], is known to be a cell-to-cell mobile mRNA. In sugarcane, DOF1 transcription factors (SsDOF1-7) have been shown to contain 1–2 cis-response elements involved in plant hormone responses, including ABA, Me-JA, and SA [58]. Our study revealed high-probability binding motifs of both PBF and MNB1A across all investigated hormonal biosynthetic and signaling genes, suggesting that these transcription factors play central regulatory roles in the plant’s innate immunity. Notably, we observed the upregulation of AHL20, PBF, and MNB1A specifically in *A. thaliana*.

### 3.3. BABA Treatment May Induce Opposing Regulatory Effects of ERFs on SA Biosynthesis in A. thaliana and H. vulgare 

In *A. thaliana*, BABA treatment led to a significant increase in the expression of SA biosynthetic genes such as ICS1, ICS2, EDS5, and WIN3, consistent with previous studies demonstrating BABA-induced SA accumulation and substantial changes in these gene levels [59,60]. Specifically, in the PAL branch of SA biosynthesis, a downregulation of the PAL gene was observed alongside the upregulation of members of the DOF transcription factor family, including MNB1A and PBF, as well as the PR1 gene. This observation aligns with findings by Yu et al. (2019), which highlighted the role of the DOF family as an upregulator of PR1 and described increased SA levels in transgenic *Arabidopsis* plants overexpressing VvDOF3, resulting in enhanced resistance to powdery mildew through the SA signaling pathway [61]. Notably, this study is the first to report the co-upregulation of MNB1A and PBF with the PR1 and NPR1 genes within the SA signaling pathway in *A. thaliana*. 

Based on the results of this study, we propose that ERF2 and ERF5 play a significant regulatory role in SA signaling. Previous research has documented the varying effects of ERF domain proteins on the SA pathway. For instance, Zhou et al. demonstrated that AtERF-1 can bind to the promoters of ICS1 and PR1, although this binding did not lead to significant changes in SA accumulation [62]. Conversely, silencing ERF2 in plants resulted in reduced SA and JA levels under biotic stress conditions [63]. The SA signaling pathway is known to interact antagonistically with the ET/JA pathways [64,65], and Xu et al. (2011) suggested that ERF proteins can coordinate with the SA pathway [66]. These findings are consistent with our results. Notably, this study is the first to report a negative regulatory role of ERF on PR1 within the SA signaling pathway in *H. vulgare*. These results imply a parallel effect on bacteria-triggered systemic immunity associated with ERFs, rather than with SA, in barley [67]. As there is no prior evidence of ERF’s role in SA regulation in monocots [68], we conclude that the BABA-induced SA response may exhibit species-specific characteristics.

### 3.4. BABA Treatment May Enhance the Expression of the JA Biosynthetic Gene LOX and JA Signaling Genes COI1, JAZ, and MYC2 by Modulating the Activity of the Master Regulatory TFs MNB1A, ERF2/5, and PBF 

The ERF family has been implicated in stress responses, facilitating broad detoxification processes involving LOXs and xenobiotic compounds [69]. In this study, BABA treatment in *H. vulgare* led to the upregulation of LOX and TIFY9 genes, which were co-upregulated with the master regulatory TF ERF2. This finding aligns with observations in peach, where trehalose alleviated chilling injury by co-upregulating TIFY9 and ERFs [70]. Additionally, microarray studies have demonstrated that JA signaling can induce the transcription of numerous ERF genes [71], with these ERFs contributing to defense mechanisms against pathogens such as Botrytis cinerea in *A. thaliana* [72,73]. Our study supports these observations by highlighting the role of ERFs as master regulatory TFs in JA signaling and biosynthesis.

Furthermore, the regulation of JA signaling was found to be influenced by the TF PBF, which interacts with COI1, JAZ, and MYC2 transcripts [74]. We observed the differential expression of these genes following BABA treatment. Consistent with Liu et al. (2018), who identified a direct interaction between PBF and MYC2 in ABA-JA signaling under abiotic stress [75], our results showed four downstream and five upstream potential binding sites of PBF in the promoter of MYC2, which was downregulated in *A. thaliana*. This suggests that BABA may inhibit JA signaling through ABA-mediated pathways.

In *H. vulgare*, we observed the upregulation of COI1 and JAZ genes in JA signaling, but no change in MYC2 expression, which followed the upregulation of the LOX and TIFY9 genes involved in biosynthesis.

Similar findings from Virág et al. (2023) demonstrated the upregulation of LOX and JAZ genes in *Hordeum vulgare* following BABA treatment [76]. These results suggest that the JA-dependent defense response is a prominent feature in *H. vulgare*’s reaction to BABA.

## 4. Materials and Methods

### 4.1. Plant Materials 

**Figure 7:**
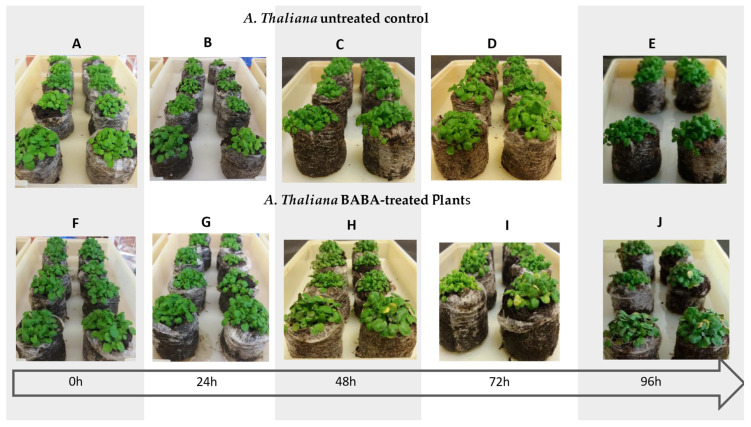
*A. thaliana* untreated control (**A**–**E**) and BABA-treated plants (**F**–**J**). Treatment concentration of BABA was 250 µM. For Ilumina RNA sequencing, samples were taken at 72 h after BABA treatment. For RT-qPCR experiments, samples were taken at 0 h, 24 h, 48 h, 72 h, and 96 h.

*H. vulgare* cv. Nure and *A. thaliana* cv. Columbia plants were cultured in a controlled environment using an MLR-352H Panasonic growth chamber (Panasonic Healthcare, Newark, NJ, USA). The temperature conditions were as follows: on the first day and night, the temperature was maintained at 23 °C. From the second day to the sixteenth day, the daytime temperature was set to 25 °C, while the nighttime temperature was lowered to 15 °C. The plants followed a photoperiod of 14 h of light, followed by 10 h of darkness, and the relative humidity was maintained at a constant level of 60 ± 5%. The priming inducer, BABA, used for treatment, was prepared in a 25 mM solution. The concentration of treatment solutions of BABA (MW: 103.121 g/mol) was 250 µM. BABA pretreatment was performed on 14-day-old plants. The treatment application was carried out using a Bürkle pressure sprayer (Bürkle GMBH, Bad Bellingen, Germany) equipped with an adjustable spray jet, with a nozzle diameter of 0.8 mm. The plant leaves were sprayed from multiple angles until they were visibly wet, ensuring complete coverage. Fresh leaves of 17-day-old plant samples were collected (72 h after the BABA treatment) for NGS sequencing. Samples were stored in DNA/RNA Shield (Zymo research, Irvine, CA, USA) at −25 °C until sequencing. Sample collection for RT-qPCR reactions were performed at 0 h, 24 h, 48 h, 72 h, and 96 h after the treatment. Sampling was carried out at 9:30 a.m. at the beginning of the daytime cycle. The samples were kept at an ultra-low temperature throughout (Figure 7).

### 4.2. Preparation of RNA-Seq Libraries

The RNA isolation and preparation of Illumina NextSeq550 libraries were performed as described by [77]. Briefly, total RNA was extracted, starting from 30 mg plant tissues and using the Direct-zol™ RNA MiniPrep System (Zymo Research, Irvine, CA, USA), according to the manufacturer’s protocol. Samples were multiplex-sequenced in the same sequencing run using dual-indexing adapters. For library amplification, adapter-selective PCR was performed. Libraries were sequenced using a single-end option, and the final output consisted of 14–26 M × 85 base pairs’ (bp) long reads (1.19–2.21 Gbp). The data availability of sequenced and processed reads was reported in Data in Brief by Hegedűs et al., 2022 [77].

### 4.3. Preprocessing and Assembly of RNA-Seq Reads

Quality control (QC), trimming, and the filtering of fastq files was performed in the preprocessing step. The QC analysis was performed with FastQC software v0.12.1 [78]. The Phred-like quality scores (Q scores) were set to >30. Poor quality reads, adapters at the ends of reads, and limited skewing at the ends of reads were eliminated by using Trimmomatic [79]. Contamination sequences and Ns were filtered out with a self-developed application GenoUtils as described earlier [80]. Reads containing Ns of more than 30% were eliminated; reads with a lower N’ ratio were trimmed to a final length >65 bp. 

### 4.4. The Mapping of RNA-Seq Reads to the Reference

Reads that passed preprocessing were further mapped to the reference genomes (GCF_000001735.4 and GCF_904849725.1) downloaded from NCBI 05.13.2023 by using Bowtie2. CDS sequences and reads that matched were kept and analyzed further. Re-annotation of the kept CDS sequences such as with Blast (Blastx, viridiplantae database), GO Mapping, GO Annotation, and functional analysis (GO-Slim) was performed. Diamond Blast alignmet was performed against the NR (10 May 2023) database, using the viridiplantae (33090) taxonomic filter.

### 4.5. Gene-Level Quantification and Analysis of Differential Expression of Genes (DEGs) 

We aligned sequencing reads to estimate gene expression from RNA sequencing experiments and kept the results in coordinates with genomic features. A count table (abundance matrix) of reads mapped to CDS sequences was created for further analysis. To identify genes expressed in significantly different quantities in distinct groups of samples, pairwise differential expression analysis was performed using the program NOISeq version 2.16.0 [81,82] that can compare samples from two experimental conditions by simulating replicates if the biological and technical replication is small. Genes that were differentially expressed between the conditions of the control and BABA treatment were identified in both species. The differential expression analysis was combined with an enrichment analysis of sample pairs. This analysis is based on the software package “edgeR” version 3.38.2 (empirical analysis of DGE in R) [83] using the statistical test GLM (quasi likelihood F-test). 

### 4.6. KEGG Pathway Analysis

Pathway analysis was performed using the KEGG database to obtain an overview of the biological mechanisms involved in the data of pairwise DEGs and to summarize the background information of molecular mechanisms. We directly linked the annotated sequences of enriched (GSEA) pairwise DEGs results to the pathways, performing an intermediate step to match a gene product to the most probable candidate found in the pathway database. Mapping was automated using omicsbox (Bioinformatics Made Easy, BioBam Bioinformatics, https://www.biobam.com/omicsbox, accessed on 3 March 2019).

### 4.7. Expression Analysis of Genes of SA, JA, ABA, and ET Biosynthetic Pathways

A total of 32 genes of the investigated pathways were selected. Orthologs of this set of genes were identified and analyzed by the DEGs analysis in *A. thaliana* and *H. vulgare* (Figure 5E). Pairwise DEGs analysis using these ortholog genes was performed in both species separately based on the abovementioned method. Results are visualized in a two-dimensional heatmap on Figure 5F,G.

### 4.8. Analysis of Master Regulatory TRFs of Genes of Biosynthesis Having Role in SA, JA, ABA, and ET Pathways

An automatic query from the EPD (https://epd.expasy.org/epd, accessed on 1 July 2023) was created by in-house software, tfcollect, v.2.0 reported by Toth et al., 2024 [84] for the investigated 32 phytohormone biosynthetic and signaling genes of *A. thaliana*. We collected all predicted TRFs with the condition of *p* < 1 × 10^−4^. Hits were further screened according to matches within the SA, JA, ABA and ET groups, thereby predicting the master regulatory transcription factors of the investigated hormonal biosynthetic and signaling pathways. After this, gene expression analysis was performed using the identified master TRF sequences of *A. thaliana* and their *H. vulgare* orthologs. The CPM (count per million) index was used for the control and BABA-treated samples for gene expression analysis. In-house software performed the automated calculation of upstream and downstream binding sites based on the PWM for the investigated genes.

### 4.9. Analysis of Relative Gene Expression by RT-qPCR

Six genes of *A. thaliana* were selected for the RT-qPCR experiments. We determined the relative expression of the genes of interest At_AAO3, At_ZEP, At_OPR1, At_AOC1, At_PAL3, and At_ICS1 and the housekeeping gene At_ACT2. The primer design was performed using the Primer3Plus (https://www.primer3plus.com/index.html accessed on 21 July 2023). Primer sequences are detailed in Table 2. GoTaq^®^ qPCR Master Mix (Promega, Madison, WI, US) was used for the qPCR reaction. Reaction cycles were as follows: 95 °C, 2.5 min; then 40 cycles of 95 °C, 20 s; 60 °C, 30 s; 72 °C, 30 s; a final cycle of 95 °C rapid heating to denature the DNA, then cooling to 55 °C. The ddCT method was used to determine relative gene expression. Reverse transcription was performed with the M-MLV reverse transcriptase enzyme (Thermo Fisher Scientific Biosciences GmbH, Leon-Rot, DE, Germany) according to the manufacturer’s recommendation, along with the necessary additional materials. Quantitative real-time PCR PerfeCTa ToughMix reagent (QuantaBio Ltd., Beverly, MA, USA) was used for qPCR reactions. Experiments were performed in three biological and three technical replicates.

## 5. Conclusions

KEGG Pathway analysis of DEGs indicated that BABA potentially activates several signaling pathways in both species, including MAPK signaling, plant–pathogen interactions, and plant hormone signal transduction. Additionally, photosynthesis was suppressed in both species. These common pathways suggest a role for stress-related phytohormones such as JA, SA, ABA, and ET in the regulatory mechanisms. Despite the identification of these shared pathways, significant differences were observed in the expression of the corresponding signaling genes, specifically the orthologs in *A. thaliana* and *H. vulgare*. To further elucidate these differences, we analyzed genes involved in phytohormone biosynthesis, signaling, and their master regulatory TFs. Using the EPD, we identified 14 master regulatory TFs with binding sites in the promoter regions of key genes related to JA, SA, ABA, and ET biosynthesis and signaling. Our gene expression studies reveal novel correlations, including the following: in ET metabolism, BABA treatment may upregulate the negative regulators EBF1/2; in ABA signaling, BABA may negatively regulate PP2C/SNRK2, potentially influenced by the master TFs AHL20, PBF, and MNB1A; and BABA might modulate ERFs with opposite regulatory roles in SA biosynthesis compared to *A. thaliana* and *H. vulgare*. These findings provide new insights into the transcriptional regulation of phytohormones during plant priming processes and suggest potential new avenues for research.

## Figures and Tables

**Table 1:** The known functions of the 14 master regulatory TFs that were screened for binding sequences in the promoter of 32 investigated genes of the JA, SA, ABA, and ET pathways.

Master TFs	TAIR ID	Function
MNB1a	AT1G51700	It is a DNA-binding protein that interacts with the 35S promoter of cauliflower mosaic virus. MNB1a seems to be a member of a multigene family. It contains the DOF domain. It may form a single zinc finger motif, and may transactivate seed storage protein genes in developing seeds [31].
PBF	AT3G19290	PBF (ABF) is a bZIP transcription factor. It activates the ABA-inducible expression of LTI65/RD29B. It binds specifically to the ABA-responsive element (ABRE), and acts through SnRK2 pathway [32].
DOF2	AT3G21270	DOF2 (ADOF2) encodes a DOF zinc finger protein DOF2. It is preferentially expressed during cell growth. It may have redundant functions with OBP4 regarding cell growth regulation [33].
DOF2.4	AT2G37590	DOF2.4 encodes the DOF zinc finger protein PEAR that may concentrate in the peaks of protophloem sieve elements, activating the expression of genes involved in radial growth. DNA binding occurs with one finger [34].
DOF5.3	AT5G60200	It encodes a DOF-type transcription factor. The protein PEAR may concentrate in the peaks of protophloem sieve elements, activating the expression of genes involved in radial growth. It has been reported in the preprocambial stage in leaves, suggesting their role in preprocambial development [35].
AHL20	AT4G14465	It is an AT-hook protein. Its overexpression results in early flowering in short and long days. It binds AT-rich DNA sequences related to the nuclear matrix attachment regions (MARs) (by similarity), and negatively regulates plant innate immunity (PTI) to pathogens by downregulating PAMP-triggered NHO1 and FRK1 expression [36].
AGL55	AT1G60920	It is a MADS-box family protein, a positive regulator of flowering. It may play a role in root development [37].
REF6	AT3G48430	It encodes a Jumonji N/C and zinc finger domain-containing protein that acts as a positive regulator of flowering in an FLC-dependent pathway. It may play a role in brassinosteroid signaling by affecting histone methylation in the promoters of BR-responsive genes. It is involved in the transcriptional regulation of hundreds of genes regulating developmental patterning and responses to various stimuli [38].
ERF2	AT5G47220	It encodes a member of the ERF (ethylene response factor) subfamily B-3 of the ERF/AP2 transcription factor family (ATERF-2). The protein contains one AP2 domain, and functions as an activator of GCC box-dependent transcription. It is a positive regulator of JA-responsive defense genes and resistance to *F. oxysporum,* and enhances JA-based inhibition of root elongation [39].
ERF5	AT5G47230	It encodes a member of the ERF (ethylene response factor) subfamily B-3 of the ERF/AP2 transcription factor family (ATERF-5). It is involved in regulating gene expression by stress factors and components of stress signal transduction pathways. The mRNA is cell-to-cell mobile. It may play an important role in plant innate immunity, regulating chitin and other plant defense pathways in response to different pathogens [40].
OBP3	AT3G55370	It encodes a nuclear-localized DOF domain-containing transcription factor expressed primarily in roots, and is responsive to salicylic acid. Transgenic overexpressors have yellow leaves and short, defective roots [41].
PEAR2	AT5G02460	It encodes a PEAR protein involved in the regulation of radial growth. It is probably involved in early processes for vascular development [42].
DOF5.8	AT5G66940	The overexpression of DOF5.8 modulates the auxin response, impairing vein formation in *A. thaliana*. It regulates its direct target gene ANAC069 positively, under abiotic stress conditions such as the presence of salt, drought, and ABA treatment [43].
HMG I_Y	AT1G14900	It binds A/T-rich DNA with a highly dynamic distribution in the nucleus. It is one of the HMGA proteins affecting the local chromatin structure in several ways, including the bending, straightening, unwinding, and looping of substrate DNA. The biological function of plant HMGA proteins is not known [44].

**Table 2:** The used primer pairs of *A. thaliana* genes for RT-qPCR validation.

Gene	Primers	Product Size	Plant Hormone	Melt Peak (°C)
At_AAO3	F: TGGGAAGCTCACAGCACTGG	182	ABA	83
	R: CGGGAGCTCTCATCGCTGTT			
At_ZEP	F: CGATGACCGGCTTCGAGAGT	152		81.5
	R: TTCCGACGATGCAAGGTTGA			
At_OPR1	F: CTCAGCCTCACGCTGCCATA	151	JA	82.5
	R: ATTGGCTTCCATGCCTCCAC			
At_AOC1	F: GGCTGGTCTCTGCGTTGTGA	162		83.5
	R: ATTCCAGCGCCACCAGTGAT			
At_PAL3	F: CCAAAGCGCAGAGCAGCATA	195	SA	83
	R: TTCGCCACCTGACTCACTGC			
At_ICS1	F: TGACCAGCAAATCGGAGCAA	197		84
	R: CTGCAGAGCCGATACCAGCA			

## Data Availability

The data of the DEGs are available at Mendeley Data in the folder ‘DEGs of BABA treatment AT HV’. The data of the TFs investigated and the 32 genes of phytohormone biosynthesis are available in the folder ‘A. thaliana-0.0001.zip’. Virág, Eszter (2024), “Phytohormone biosynthesis and signalling in *A. thaliana* and *H. vulgare*”, Mendeley Data, V1, doi: 10.17632/mkjrs4cjxk.1 [85].

## Supplementary Materials

The supporting information can be downloaded at https://www.mdpi.com/article/10.3390/ijms25179179/s1.
