# Supplementary material for: Master Regulatory Transcription Factors in β-Aminobutyric Acid-Induced Resistance (BABA-IR): A Perspective on Phytohormone Biosynthesis and Signaling in Arabidopsis thaliana and Hordeum vulgare
Source: Int J Mol Sci. 2024 Aug 23;25(17):9179. doi: 10.3390/ijms25179179 (PMC11395473; doi:10.3390/ijms25179179)
Supplement: Supplementary file 1 [file ijms-25-09179-s001.zip › ijms-3163621-supplementary.pdf]

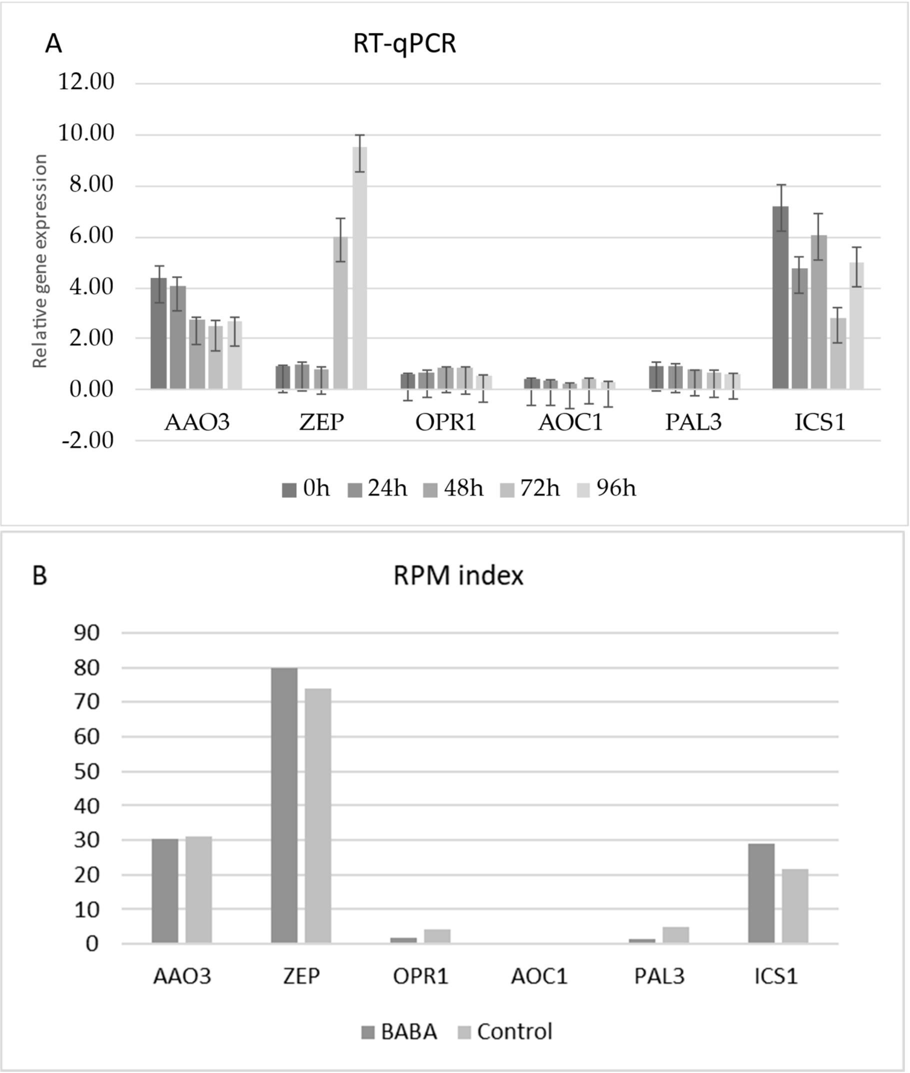

Figure S1 Correlation analysis of genes of interest. The relative gene expression determined by RT-qPCR and RPM index of AAO3, ZEP, OPR1, AOC1, PAL3 and ICS1 were used for the correlation. R= 0,996249. Relative gene expression values of genes of interest were determined by RT-qPCR method (A). RPM indexes of the genes of interest were determined from the in silico data at 72h samples (B).
